# Origin of the ornamented *bâton percé* from the Gołębiewo site 47 as a trigger of discussion on long-distance exchange among Early Mesolithic communities of Central Poland and Northern Europe

**DOI:** 10.1371/journal.pone.0184560

**Published:** 2017-10-04

**Authors:** Grzegorz Osipowicz, Henryk Witas, Aleksandra Lisowska-Gaczorek, Laurie Reitsema, Krzysztof Szostek, Tomasz Płoszaj, Justyna Kuriga, Daniel Makowiecki, Krystyna Jędrychowska-Dańska, Beata Cienkosz-Stepańczak

**Affiliations:** 1 Institute of Archaeology, Nicolaus Copernicus University, Toruń, Kuyavian-Pomeranian Voivodeship, Poland; 2 Department of Molecular Biology, Medical University of Lodz, Łódź Voivodeship, Poland; 3 Institute of Zoology, Jagiellonian University, Kraków, Lesser Poland Voivodeship, Poland; 4 Bioarcheology and Biochemistry Laboratory, University of Georgia, Athens, Georgia, United States of America; University at Buffalo - The State University of New York, UNITED STATES

## Abstract

This article describes evidence for contact and exchange among Mesolithic communities in Poland and Scandinavia, based on the interdisciplinary analysis of an ornamented *bâton percé* from Gołębiewo site 47 (Central Poland). Typological and chronological-cultural analyses show the artefact to be most likely produced in the North European Plain, during the Boreal period. Carbon-14 dating confirms the antiquity of the artefact. Ancient DNA analysis shows the artefact to be of *Rangifer tarandus* antler. Following this species designation, a dispersion analysis of Early-Holocene reindeer remains in Europe was conducted, showing this species to exist only in northern Scandinavia and north-western Russia in this period. Therefore, the *bâton* from Gołębiewo constitutes the youngest reindeer remains in the European Plain and south-western Scandinavia known to date. An attempt was made to determine the biogeographic region from which the antler used to produce the artefact originates from. To this end, comprehensive δ^18^O, δ^13^C and δ^15^N isotope analyses were performed. North Karelia and South Lapland were determined as the most probable regions in terms of isotopic data, results which correspond to the known distribution range of *Rangifer tarandus* at this time. In light of these finds, the likelihood of contact between Scandinavia and Central Europe in Early Holocene is evaluated. The *bâton percé* from Gołębiewo is likely key evidence for long-distance exchange during the Boreal period.

## Introduction

In Prehistoric communities, exchange of gifts constituted a common element in all contacts, including both inter-personal and inter-group contacts. Seemingly voluntary and disinterested, gift exchange actually involved an obligation to reciprocate and, oftentimes, economic interests [1]. In archaeological perspective, interpreting exchange or the phenomenon of a gift in Prehistoric communities lies in studying artefacts and raw materials, such as stone transported over substantial distances due to the technological significance, or prestigious items such as metal products, often deposited in graves. Much information also can be gained by specialist and multidisciplinary studies on single and seemingly ordinary artefacts.

**Fig 1:**
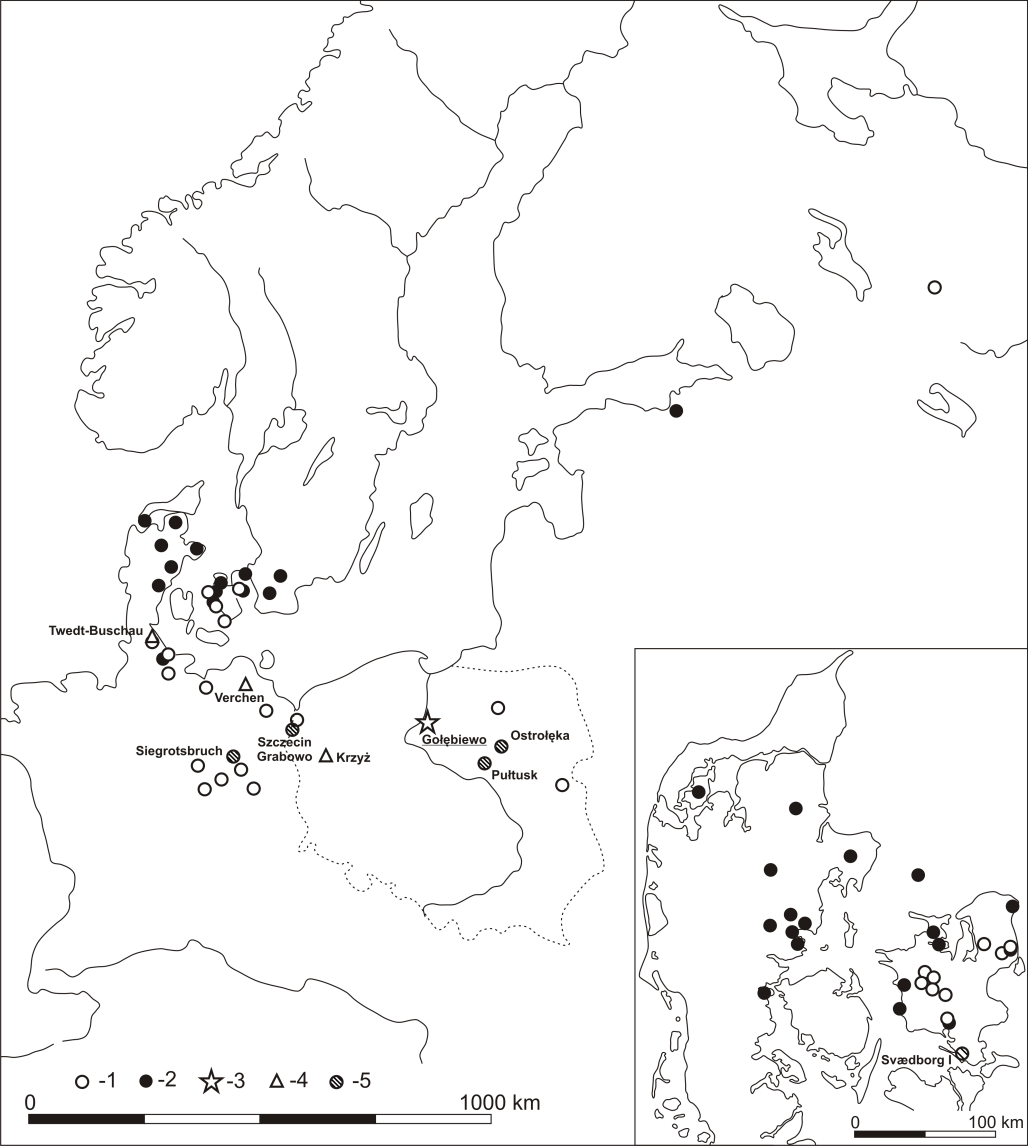
Location of Gołębiewo 47 site and other finds of ornamented and zoomorphic bâtons percés in Europe (after [11], with the authors’ amendments): 1—Ornamented bâtons percés from the Boreal period and the Boreal/Atlantic transition, 2—Ornamented bâtons percés from the Atlantic period, 3—Ornamented bâtons percés from Gołębiewo; 4 –Artefacts stylised on the elk's head; 5 –Artefacts ornamented with hatched triangles.

In spring 2013, in village named Gołębiewo situated in the northern part of the Chełmno-Dobrzyń Lake District (Central Poland–Fig 1) during an excavation of a fish pond, an ornamented item made of antler was found. Though substantially processed, the material was tentatively assigned as reindeer antler. The Mesolithic style and form of the artefact gave rise to a number of questions and concerns, including species identification, antiquity, place of origin, and place of production. Considering the substantial intellectual value of the find for interpreting prehistoric exchange, to address these issues, the artefact was subjected to a multidisciplinary analysis that considered its stylistic and cultural attributes, radiocarbon dates and DNA studies to determine antiquity and species, and stable carbon, nitrogen, and oxygen isotope analysis to estimate geographic place of origin of the animal. Results point to the possible existence of contact and exchange between the people of Central Poland and those inhabiting north-eastern Scandinavia in the Early Mesolithic.

On account of the discovery of the incised reindeer antler artefact, excavations were carried out at Gołębiewo in 2015. These excavations did not yield any other prehistoric objects. A surface survey was conducted in the immediate vicinity of the site, which also yielded no evidence of Mesolithic settlement. Generally, Mesolithic settlements are poorly recognized in the Chełmno-Dobrzyń Lakeland. Until recently, there were only a few dozen sites from this period known [2], and of these, only a few were subjected to detailed surface surveys or excavations [3–5].Of note, however, is the discovery of graves dated to the Boreal period at Mszano, site 14 [4] and subsequent interdisciplinary spatial studies conducted on a complex of well-preserved late Mesolithic sites near the villages of Sasieczno and Ludowice [6–10]. Since 2017 the studies on Mesolithic settlement in region are funded by the National Science Center of Poland and carried out as part of the project titled *Mesolithic Societies of the Chełmno-Dobrzyń Lakeland–daily life*, *mobility*, *external contacts and relations with the natural environment* (award number 2016/23/B/HS3/00689).

## Material and methods

### Description of the artefact

**Fig 2:**
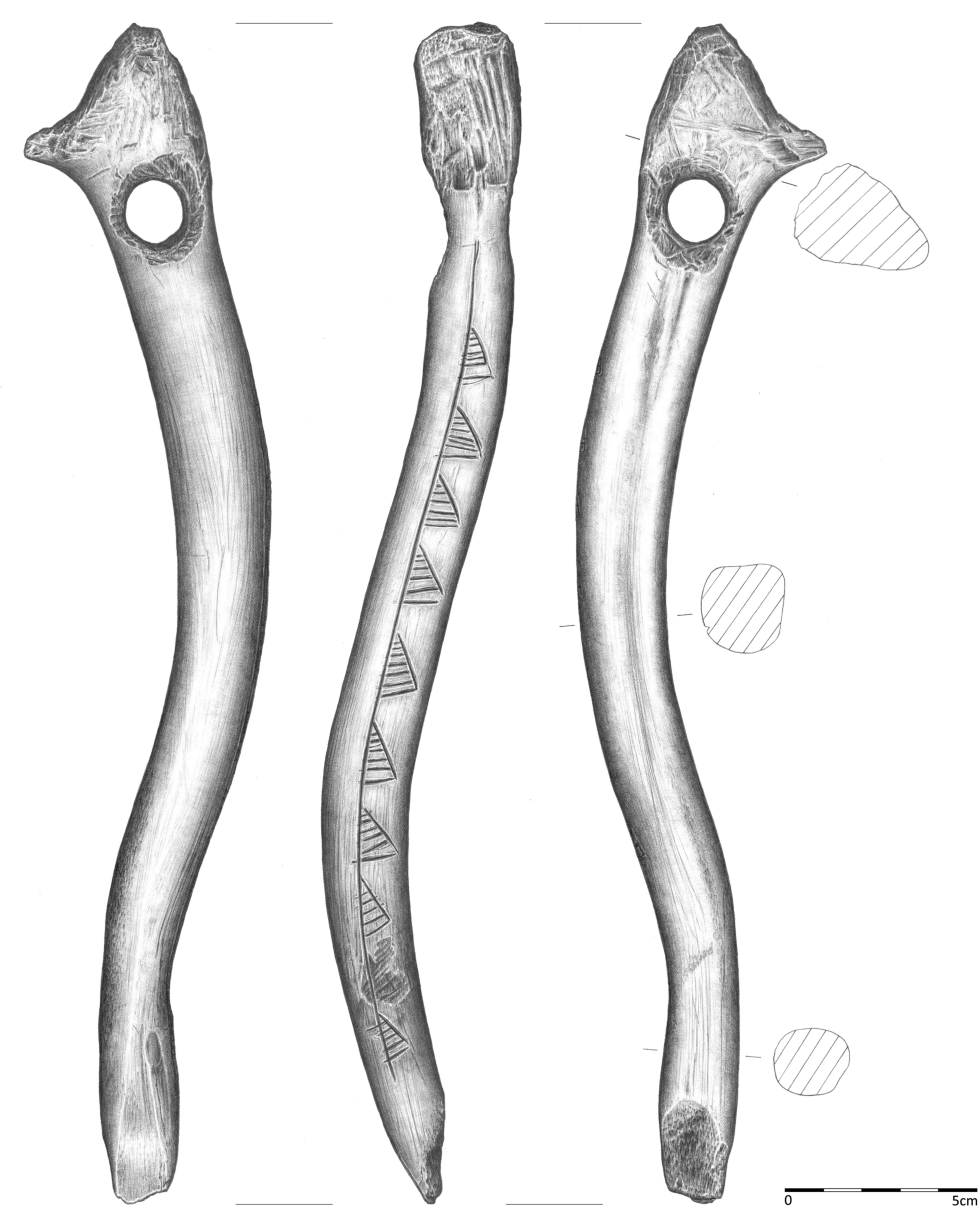
Bâton percé from Gołebiewo 47 (draw. B. Kowalewska).

The item excavated in Gołębiewo, referred to in the literature as a *lochstab/ bâton percé*, *kommandostab/ bâton de commandement*, a mattock or a shaft, is 30.5 cm long with an oval cross-section and a diameter of approximately 1.8–2.0 cm at the base, 2.1–2.4 cm in the middle part and up to 2.9 cm near the perforation (Fig 2). The artefact was made in a manner similar to other products of this sort from Europe [11]. The formation of the proximal part (the head) required removal of the burr and drilling of a round hole of ab. 16 mm in diameter and a slightly hourglass-like cross-section about 5 cm from the tip. The precisely processed head was formed with a shape of a dull concave blade ab. 2.3 cm in width, positioned perpendicularly to the line of the perforation, on the symmetry axis of the specimen. Just above the perforation, on one of the lateral surfaces, an approximately 1.5 cm long dull spike was formed from a piece of a brow tine. The symmetry of two parts of the head defined by a line running through the brow tine spike along the axis of the product indicates that this part of the artefact was made with exceptional accuracy. The described elements were likely intended by the craftsman to form one non-accidental whole or to constitute some sort of a zoomorphic stylisation (more information below). The way in which the lower part of the item was formed is difficult to determine due to a substantial damage.

**Fig 3:**
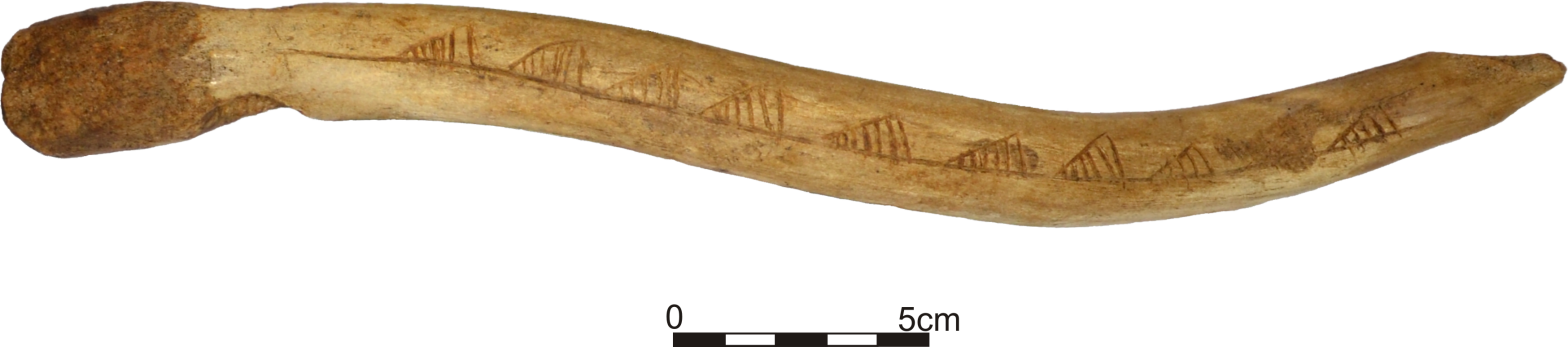
Bâton percé from Gołebiewo 47 –ornamentation (Photo J. Kuriga).

The ornaments on the backside (backed edge) of the artefact (Figs 2 and 3), i.e., the surface opposite to the spike formed on the head, were made in a horizontal arrangement along the axis of the product by cutting or carving. The ornaments cover an area from the drilled perforation to the point located approximately 3 cm above the base of the *bâton percé*. Originally, the ornaments covered 10 asymmetric triangles with oblique striations similar in shape and size, situated in a horizontal line, the so-called triangles on a line, filled with oblique shading [12]. The second lowest triangle was partially removed (by whittling). All triangles are filled by 4 to 6 incisions (mostly 5) that are parallel to the shortest side. The artefacts is permanently and publicly deposited in Fr. dr Władysław Łęga Museum in Grudziądz (inv. no. MGA7838).

## Methodology of paleobiological studies

### DNA sequence analysis

Two fragments of the studied artefact were transferred in sterile containers and delivered to the Department of Molecular Biology, Medical University of Łódź (sample lab. ID: PKX3). Each fragment was firstly cleaned mechanically and subsequently washed in 5% NaClO and in 96% ethanol. After 30-minute exposition to UV light, each sample was powdered in a freezer mill SPEX Sample Prep 6770. The thus obtained powder (0.29 g and 0.32 g) was suspended in 0.5 M EDTA, pH = 8.0 and incubated for 48 hours. Afterwards, proteinase K and PTB were added and incubated at 56°C for the next 2 hours. Subsequently, the obtained solution was submitted to DNA isolation in MagNA Pure® Compact Nucleic Acid Purification System (Roche).

Sample processing was carried out in a laboratory dedicated to working with ancient molecules. The DNA of contemporarily living *Rangifer tarandus* and *Cervus elaphus* representatives was isolated and processed in a separate lab space. Moreover, mock samples were followed in order to avoid contamination with modern DNA. Instead of cloning, sequencing of two separate isolates from the same specimen was applied to confirm reproducibility of the obtained data, as suggested by Winters et al. [13].

A fragment of cytochrome B gene with a relatively high number of SNPs differing *Rangifer tarandus* and *Cervus elaphus* species has been chosen, producing 144 bp and 121 bp long PCR products, respectively (Table 1). The specificity of carefully designed primes differentiating species was verified using DNA isolated from skeletal material of contemporary living representatives of both species and then used to target sequences of the ancient samples’ DNA.

**Table 1:** Primers, annealing temperature and length of PCR products used in differentiation.

mtDNA Fragment	Primer pairs	Annealing temp. [˚C]	Product length [bp]
*Rangifer tarandus* (14282–14425)	CTACAAATCCTTACCGGTCT	57	144
	AAATATTGATGCTCCGTTG		
*Cervus elaphus* (14340–14460)	TAACAGCATTCTCCTCTGTC	57	121
	CGCCCTACATGTATGAATAG		

### Stable isotopes analysis

A small piece of antler was demineralised in 0.2 M hydrochloric acid (HCl) for several days, rinsed, and soaked in 0.125 M sodium hydroxide (NaOH) to remove humic contaminants. The specimen was dissolved in 10^−3^ M HCl, filtered, and freeze-dried. The dried residue was homogenised in an agate mortar and pestle, 0.546 mg weighed into a tin capsule, and combusted and analysed in a Costech ® Elemental Analyzer coupled to a Finnegan MAT 252 IR-MS housed at the University of Georgia Centre for Applied Isotope Studies (sample lab. ID: SIEL S.O.#317-Reindeer). Both δ^13^C and δ^15^N values are given as per mille values (‰) reported according to the equation [δ = (R_sample_−R_standard_) / R_standard_ x 1000]. Results of replicate analyses of the acetanilide standard were 0.07‰ and of the internal spinach standard were 0.06‰ for δ^13^C; results of replicate analyses of the internal bovine standard were 0.07‰ for δ^15^N. Results are reported to the nearest 0.1‰.

The analysis of the oxygen isotope composition was conducted with regard to bone apatite phosphates (Insitute of Zoology, Jagiellonian University in Krakow–sample lab. ID: G01). The analytic isolation of these phosphates was conducted according to O’Neil et al. [14]. After washing bone fragments in distilled water using an ultrasonic cleaner, the material was dried and then ground in a ball mill (Retsch MM 200). Sodium hypochlorite (NaOCl) was added to 0.4 mg powdered bone sample. After a 24-hour incubation, it was rinsed and sodium hydrate was added, and later used to incubate the material for 48 hours. When the humic substances and other substances had been removed, the bone powder was incubated in hydrofluoric acid for 24 hours. The apatite dissolved in acid was neutralised using potassium hydroxide and, after having added silver nitrate buffer, it was incubated at 70°C. The crystallised silver phosphate, filtered, dried and weighed (250± 20 μg), was then placed into silver capsules. The isotopic composition was determined using mass spectrometer IsoPrime, coupled with elemental analyser EuroVector (Department of Radioisotopes, Silesian University of Technology in Gliwice). The delta notation (δ^18^O_sample_ = [(R_sample_-R_standard_)/R_standard_]*1000) for samples referred to the value of isotopic composition for the laboratory standard NIST 120c was presented on the VSMOW scale having been standardised to clean ocean water (δ^18^O value for the standard NIST 120c – 21.7‰, measurement uncertainty– 0.17‰.). In order to determine the local standard oxygen isotope composition in the area of Gołębiewo, the Online Isotopes in Precipitation Calculator (OIPC) was applied in the study.

To attempt to identify the place of origin of the antler on the basis of oxygen isotope data, using the database of the Global Network of Isotopes in Precipitation (GNIP) [15] created by the International Atomic Energy Agency (IAEA) and the World Meteorological Organisation (WMO), the composition of the artefact was compared with referential δ^18^O_w_ values in probable locations of origin of the antler used for making *bâton percés*, including Norway, Denmark, Sweden, Finland, the Kola Peninsula, Russia, Estonia, Latvia, as well as Poland. Moreover, results of isotopic analyses from Lapland-Rovaniemi [16], southern, northern and eastern parts of Finland [17] were also taken into consideration. With the data concerning monthly average isotopic composition of precipitation water computed for Gołębiewo, the said values were converted into values of isotopic composition of bone phosphates (δ^18^O_p eq.conversion_) with conversion error taken into account, using the following conversion equation: δ^18^O_W_ = 1.73 (±0.21) × δ^18^O_P_ − 37.25 (±3.55) from a study by Daux et al. [18]. The said equation was also applied in calculations where output data derived from the above-mentioned papers. In majority of the areas and locations accounted for in this paper GNIP monitoring stations are situated: Gołębiewo (Poland), Lista (Norway), Taastrup (Denmark), Gothenburg, Stockholm, Umea, Ricklea (Sweden), Espoo, Kuopio, Talvivaara, Kuusamo, Rovaniemi (Finland), Moscow, Saint Petersburg, Arkhangelsk (Russia), Tartu, Vilsandi (Estonia), Riga (Latvia). In the case of Gołębiewo, Kuusamo and Talvivaara, where the isotopic composition of precipitation water is no longer measured, their delta values were estimated using the OIPC calculator.

## Results and discussion

### Chronology and cultural origin of the artefact from Gołębiewo

**Fig 4:**
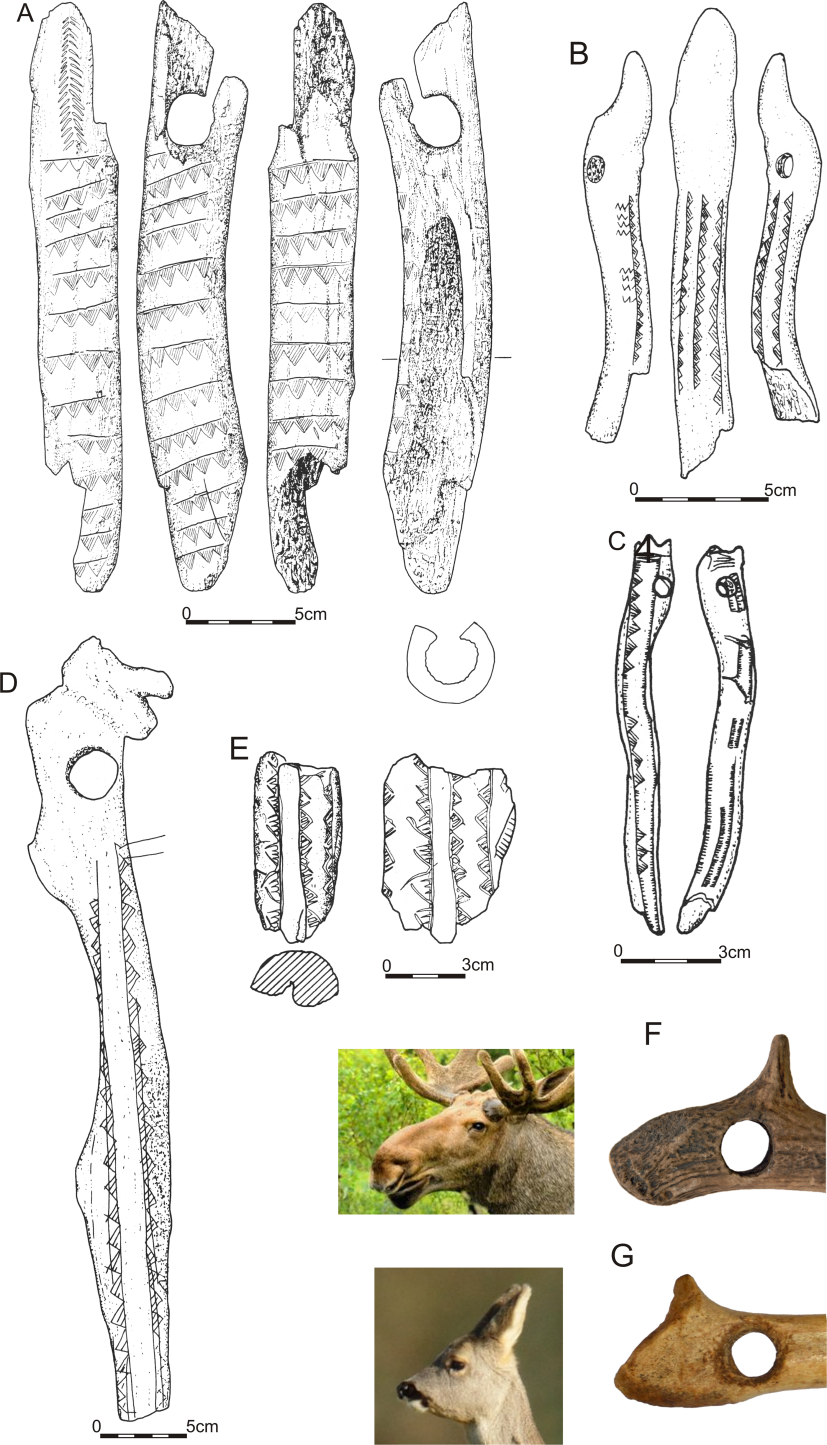
Artefacts ornamented with hatched triangles (1–5) and a comparison of bâtos percé heads from Krzyż Wielkopolski 7 and Gołębiewo 47 sites with animal heads they might depict (6, 7): A–Pułtusk (after [25]), B–Ostrołęka (after [24]), C–Szczecin-Grabowo (after [26]), D—Svædborg I (after [11]), E–Siegrotsbruch (after [27], F–Krzyż Wielkopolski 7 (after [36]), G–Gołębiewo 47.

Products of the *bâton percé* kind appear as early as in the Magdalenian culture (i.a. [19–21]). However, considering its manufacture, its engraving and the geographic location of the Gołębiewo site, it can best be associated with early Holocene hunter-gatherer groups. The *bâtons percés* of the early Holocene were made in a standardised manner with antler of large deer, and by fashioning the head with a cutting edge or blade [11]. However, objects of this type can be generally classified chronologically due to key differences; for example, size, diameter, or the method of making holes. Artefacts dated to the Boreal and the Early Atlantic tend to be 40–50 cm long and about 3.3–4.3 cm wide. Atlantic specimens are longer, usually from 50 to 70 cm long and narrower (2.7–4 cm wide on average), and with smaller holes [11]. Key differences pertain to the engravings. An motif situated vertically along the axis of the artefact made by carving with various geometric patterns is typical of the Boreal and the Early Atlantic period of the Mesolithic. In the Atlantic, many different types of incisions and piercing are dominant, whereas geometric motifs are very rare. No product from that period involves a motif with a geometric pattern arranged in one line [11]. Thus, the depicted item can be dated to the Boreal or the Early Atlantic with high probability. Further arguments supportive of such a chronology are provided by the analysis of the recorded ornaments. According to J. G. D. Clark’s [12] classification, a pattern involving triangles on a line, filled with oblique shading is an element of the second category (geometric motives) type “l”. This motif, noted, for example, on the site in Svaerdborg, can be related to the Maglemose Culture and dated to the Boreal. According to the researcher, it is typical of communities of the Maglemose complex located in Zealand and Poland, and separates both these regions (along with other types of ornamental work overlapping in this area), as well as Jutland, Holstein and Sweden from neighbouring areas. A similar view is presented by Tomasz Płonka, who classified this type of engraving as type E4, noting that items decorated in this manner may be dated to the Boreal and in some regions (including Poland) also the Early Atlantic [11]. The distribution of these products reaches to the north-eastern regions of the present-day Poland, though it is the first artefact of this sort in the Lake District of Chełmno and Dobrzyń (Fig 1). The other specimens with this type of engraving (stray finds–Figs 1 and 4), excavated at Polish sites are from areas nearby Szczecin-Grabowo [11,22,23], Ostrołęka [11,12,23,24] and Pułtusk [11,25]. This type of engraving is rare outside Poland, recorded only on two artefacts: a partially preserved specimen from Siegrotsbruch nearby Brandenburg and another at Svaedborg 1 site.

In order to determine the relative chronology of the artefact and the location where it was produced, one ought to take a closer look on the way its proximal part (the head) was formed. It was suggested above that it might constitute a zoomorphic stylisation. There are a number of this type of product of Early-Holocene mobile art; for example, a depiction of a bird and a fish from Nizhneye Veretye [28], birds from Veretye 1, Ivanovskoe 7 and Lepaa [29–31], serpent and elk heads from Oleny Island [32], serpent from Tõrvala [33] or elk heads from Zamostje 2 [34,35]. There are also zoomorphically stylised *bâtons percés* from the area of Central European Plains, dated to the Boreal period of the Mesolithic. Recently, this type of zoomorphic artefact was excavated at Krzyż Wielkopolski 7 site [36], whereas former specimens come from the area of Northern Germany, specifically Twedt-Buschau [36] and Verchen [37]–Fig 1. In all these products, heads present a uniform depiction of an elk head (Fig 4F; [36]). The Gołębiewo find is different from these other examples in the presence of engravings, the way in which the proximal part was formed, and the location of the drilled hole. However, shared elements call for attention, particularly a spike formed of a brow tine, which in all the above-mentioned cases imitates an ear. The presence of this element in the *baton* from Gołębiewo together with its scrupulously processed head and its shape suggest that the creator had a similar intent. The artefact does not depict an elk head, differing in this regard to the other artefacts listed here. Nevertheless, it might constitute a simplification of a depiction of a head of another mammal, e.g., a roe deer or a red deer (Fig 4G), whereas the schematic manner of the depiction might be intentional (cf. [31]). This hypothesis should be scrutinized, though results of the performed radiocarbon dating seem to support it. The *bâtons percé* from Gołębiewo is dated 8790 ± 50 years BP (Poz-73613), which situates it in an exceptionally short chronological span determined by the above mentioned zoomorphic artefacts (Table 2). Importantly, in a chronologically corresponding site in Verchen, where one of the items depicting an elk head comes from, a bone and a damaged *bâton percé* specimen were found, both decorated with a vertically positioned hatched triangles [37], similar to the one visible on the object from Gołębiewo. To conclude, both the characteristics of the analysed product and the corresponding carbon dating suggest it was made in the Central European Plains in the Boreal period of the Mesolithic.

**Table 2:** ^14^C dates cited in work. Calibration with the OxCal 4.2 program.

Locality	Direct dated			Sample material	Citation
	Lab. Code	14C BP	Range cal. BC (1s)		
Gołębiewo, Poland	Poz-73613	8790 ± 50	7956–7750	Antler, *Rangifer tarandus*	-
Verchen, Germany	UtC 9739	8820± 60	8171–7756	Antler	[37]
Krzyż Wielkopolski, Poland	Poz-27419	8980± 80	8284–7988	Antler	[38]
Twedt-Buschau, Germany	Poz-31269	9130± 50	8426–8279	Antler	[36]
Maszycka Cave, Poland	Ly-2454	15490±310	17149–16450	No data	[39]
Maszycka Cave, Poland	Ly-2453	14520±240	16033–15442	No data	[39]
Deszczowa Cave, Poland	Gd-9464	16150±280	17894–17196	Layer VIIIa	[40]
Deszczowa Cave, Poland	Gd-10212	17480±150	19394–18948	Layer VIII	[40]
Deszczowa Cave, Poland	Poz-3751	19250±120	21424–21066	Layer VIII	[41]
Deszczowa Cave, Poland	Poz-5195	15850±120	17308–16995	Layer VIIa	[41]
Deszczowa Cave, Poland	Poz-3750	12860±110	13596–13236	Layer VIIa	[41]
Deszczowa Cave, Poland	GO-10212	17480±150	19394–18948	No data	[42]
Kraków Spadzista street, Poland	Poz-1248	23750±140	25986–25746	Layer III, 6a	[42]
Kraków Spadzista street, Poland	GrN-6636	23040±170	25563–25265	Layer III, 6a	[42]
Kraków Spadzista street, Poland	OxA-635	20200±350	22864–21938	Layer 6a	[42]
Kraków Spadzista B "Workshops", Poland	Ly-2542	21000±900	24205–22224	Layer 6, base, 6a	[42]
Mamutowa Cave, Poland	not available	20260±250	22758–22068	Layer VI	[43]
Mamutowa Cave, Poland	Gd-10021	20260±250	22758–22068	Level 6	[42]
Mamutowa Cave, Poland	Gd-10024	11650±200	11761–11350	Level 2	[42]
Chmielewo, Śniardwy Lake, Poland	Poz-4162	10060±50	9798–9458	Antler, *Rangifer tarandus*	[44]
Oblazowa Cave, Poland	Poz-1134	13800±70	14899–14595	Layer VII	[41]
Oblazowa Cave, Poland	Poz-3741	12830±70	13460–13215	Layer VII	[41]
Oblazowa Cave, Poland	Poz-1132	11260±60	11225–11116	Layer II	[41]
Stellmoor, Germany	K-4580	9810±100	9441–9158	*Rangifer tarandus*	[48]
Rottenburg-Siebenlinden 1, Germany	ETH-8265	9110±80	8437–8252	*Rangifer tarandus*	[45]
Rottenburg-Siebenlinden 1, Germany	Gra-39961	9225±35	8535–8347	*Rangifer tarandus*	[49]
Kitley Shelter Cave, UK	OxA-7142	9670 ± 110	9256–8844	*Rangifer tarandus*	[51]
Aveline’s Hole, UK	OxA-802	9670 ± 110	9256–8844	*Rangifer tarandus*	[51]
Darent Gravels, UK	BM-1674	9760 ± 70	9298–9176	*Rangifer tarandus*	[51]
Gough’s Cave, UK	BP Q-1581	9920± 130	9664–9266	*Rangifer tarandus*	[51]
King Arthur's Cave, UK	OxA-6839	9930 ± 90	9652–9288	*Rangifer tarandus*	[50]
Risbanke, Denmark	K-7074	9180 ± 80	8526–8296	*Rangifer tarandus*	[52]
Dumpokjauratj, Sweden	Ua-19212	8630± 85	7750–7576	Charred wood	[57]
Kangos Raä 22, Sweden	Ua-23266	8555± 65	7609–7528	Burnt bone, *Rangifer tarandus*	[56]
Kangos Raä 22, Sweden	Ua-23818	8720± 60	7814–7608	Burnt bone, *Rangifer tarandus*	[56]
Kitkiöjavri, Sweden	Ua-24560	8055± 55	7081–6830	Burnt bone, *Rangifer tarandus*	[56]
Kunda Lammasmägi, Estonia	UA-3005	9330± 120	8751–8352	Bone, *Alces alces*	[54]
Lake Vasula, Estonia	TA-1958	10670± 80	10745–10625	Bone, *Rangifer tarandus*	[54]
Lake Kunda, Estonia	Hela-597	10170± 95	10090–9674	Antler, *Rangifer tarandus*	[54]
Lake Kunda, Estonia	Hela-598	9970± 85	9655–9318	Antler, *Rangifer tarandus*	[54]
Tetele, Latvia	Hela-608	10345± 75	10434–10110	Bone, *Rangifer tarandus*	[54]
Lake Lubãns, Latvia	Hela-608	9980± 70	9654–9331	Bone, *Rangifer tarandus*	[54]
Zvejniki II, Latvia	Ua-1979	9170± 70	8458–8295	Harpoon, *Rangifer tarandus*?	[60]
Rudamina, Lithuania	Hela-600	10435± 95	10576–10202	Antler, *Rangifer tarandus*	[54]
Malmio 1, Finland	Ua-41079	8910± 64	8228–7972	Burned bone, *Rangifer tarandus*	[68]
Enontekiö, Finland	Hel-2564	7750± 120	6746–6452	*Rangifer tarandus*	[53]
Inari, Finland	Hel-3580	7600± 90	6569–6389	*Rangifer tarandus*	[53]
Taivalkoski, Finland	Hela-27	6015± 170	5206–4719	*Rangifer tarandus*	[53]
Posio, Finland	Su-2681	5750± 110	4716–4466	*Rangifer tarandus*	[53]
Sujala, Finland	Hela-1104	8930± 85	8246–7970	Burned bone	[61]
Sujala, Finland	Hela-1103	8940± 80	8253–7972	Burned bone	[61]
Lepaa, Finland	Hela-516	7420± 75	6382–6230	Ornamented antler, *Rangifer tarandus*	[31]
Løkvika, Norway	TUa-7892	9640± 70	9226–8850	Bone bird/fish	[63]
Vylys Tom 2, Russia	LU- 7288	8690 ± 90	7908–7592	Layer 4	[71]
Vylys Tom 2, Russia	GIN-14594	8540 ± 70	7604–7524	Layer 4	[71]
Parch 2, Russia	GIN-11912	9500± 250	9229–8558	Charcoal	[73]
Parch 2, Russia	GIN-11913	9100± 250	8700–7942	Charcoal	[73]
Parch 2, Russia	GIN-11911	7800± 300	7078–6386	Charcoal	[73]
Zamostie 2, Russia	GIN-6201	7380± 60	6371–6126	Layer 8	[32]
Zamostie 2, Russia	GIN-6565	7450± 100	6417–6234	Layer 8	[32]

### Biological origin of the antler used for producing the artefact

**Fig 5:**
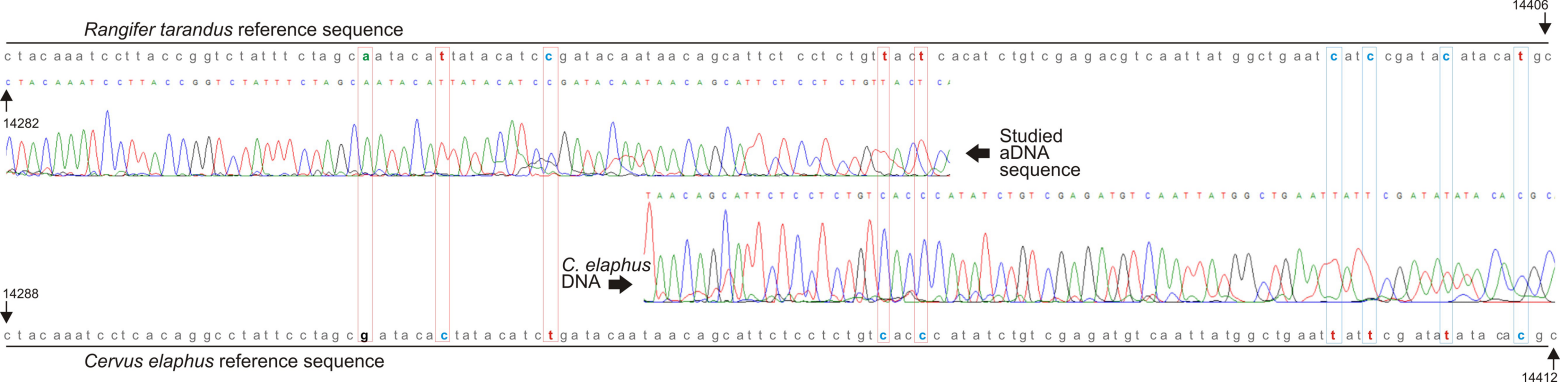
Comparison of mtDNA sequence of studied artefact with reference sequences of Rangifer tarandus and Cervus elaphus. Sites conferring origin of the sample are assigned by coloured rectangles. Details in the text.

The key objective of the conducted paleobiological research was to verify the outcome of the archaeozoological analysis, which indicated reindeer antler as the raw material used for making the artefact from Gołębiewo. Two independent attempts of DNA isolation from two separate fragments of the artefact were undertaken. Only the second one produced high enough DNA to obtain a PCR product. Only the 144 bp sequence of *Rangifer tarandus* was amplified, in contrast to the *Cervus elaphus* one (121 bp). Sequencing of the obtained fragment revealed a primary structure the deduced sequence of which was identical with the reference sequence of *Rangifer tarandus*, while 9 SNPs distinguished it from the sequence of *Cervus elaphus* (Fig 5). The obtained data strongly suggest *Rangifer tarandus* as the source of the antler used for the production of the *bâton percé*.

### Early Holocene remains of *Rangifer tarandus* in Europe

**Fig 6:**
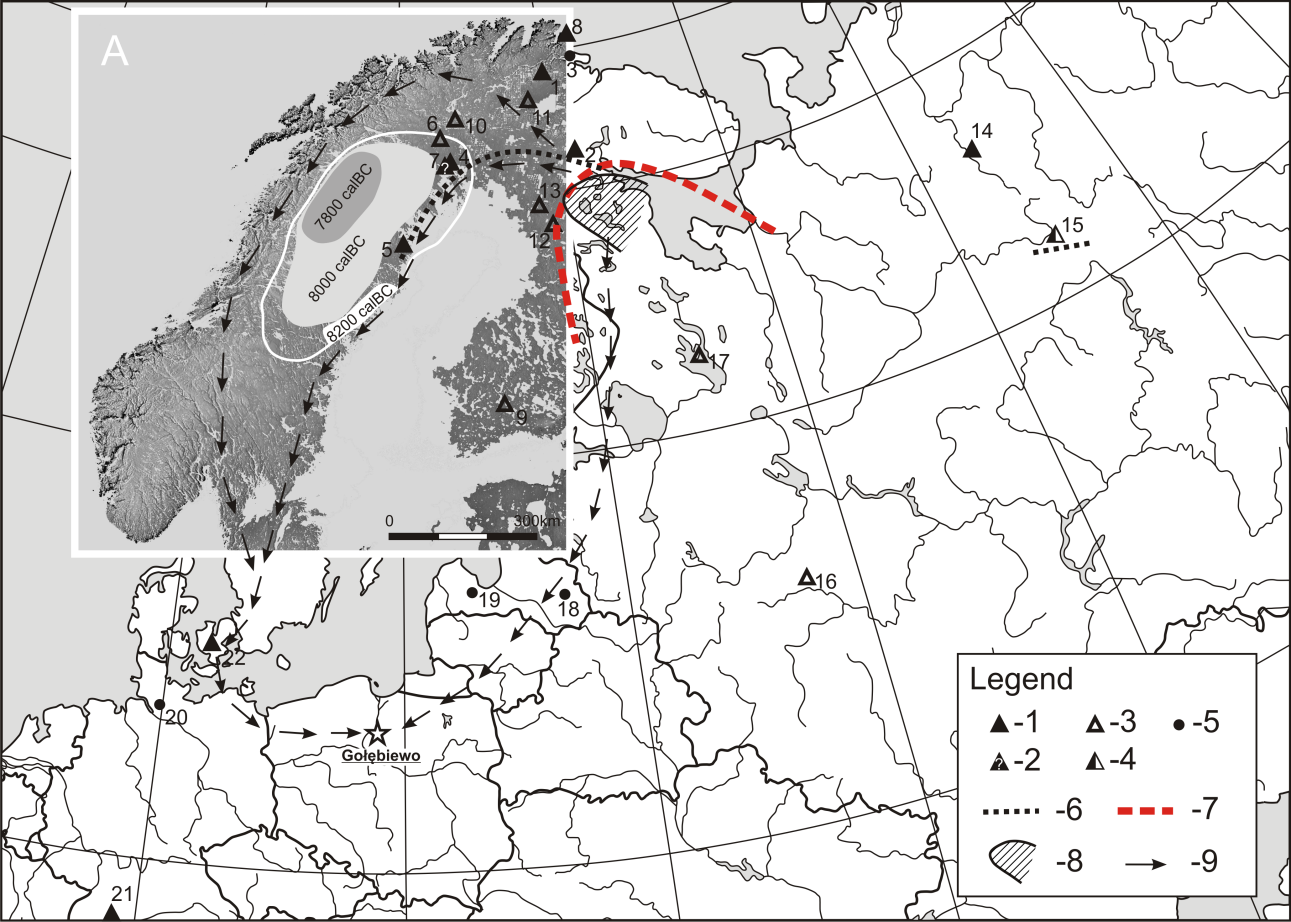
Map of the Central and North Eastern Europe with marked sites where Early Holocene remains of Rangifer tarandus were found and other significant sites mentioned in the paper. **Part A of the map presents the outline of the Scandinavian coast about 8000 cal BC and stages of the melting of ice sheet in that period (after [46,68]). The background map and the map presented in Part A are not compatible.** Legend: 1 –Rangifer bone finds of a similar chronology or older (max from the Preboreal/Boreal transition) to that from Gołębiewo; 2 –sites where Rangifer bones o of a similar chronology or older (as above) to that from Gołębiewo were probably found; 3- Rangifer bone finds of a chronology younger than that from Gołębiewo; 4 –sites where Rangifer bones of a chronology older and younger than that from Gołębiewo were found, 5 –other important sites mentioned in the paper. Sites: 1- -Sujala, 2 –Malmio 1, 3 –Fállegoahtesajeguobba, 4 –Tapuli I and II, Kongos Raä, 5 –Dumpokjauratj, 6 –Kitkiöjavri, 7 –Aareavaara sites, 8 –Løkvika, 9 –Lepaa, 10 –Enontekiö, 11 –Inari, 12 –Taivalkoski, 13 –Posio, 14 –Vylys Tom 2, 15 –Parch 2, 16 –Zamostie 2, 17 –Olenii Ostrov, 18 –Jezioro Lubãns, 19 –Zvejnieki II, 20 –Stellmoor, 21 –Rottenburg-Siebenlinden 1, 22 –Risbanke.

The vast majority of reindeer bones found in Poland are Late Pleistocene cave remains from the south regions of the country, the youngest of which were found in Obłazowa and the Mammoth Caves, both dated contextually 12 thousand cal BC (Table 2). The latest, pre-Boreal date was obtained for antler found in Chmielewo in north-eastern Poland in a chalk mine near Lake Śniardwy. This find is still about 1200 radiocarbon years older than the product form Gołębiewo. The situation in other parts of the North European Plains and southern Scandinavia is similar (Table 2, Fig 6).

As for Germany, the most recent dated remains of *Rangifer sp*. were determined for the Ahrensburg Culture site in Karstein (10900 cal BP). However, the dating was considered implausible [47,48]. The youngest (limit) chronological range of distribution of this species in the North European Plains was assumed to be defined by Stellmoor material, where the most recent excavated remains were dated to the second half of the 10^th^ millennium cal BC [48]. A single find potentially important to the studies on the artefact from Gołębiewo is a reindeer bone from Rottenburg-Siebenlinden 1 site, which was found in the context of ecofacts and products of typical Holocene provenance [49,50]. Both radiocarbon dates obtained for this item indicate the Boreal, whereas the very depositional context suggests a kind of import [48]. The youngest plausible finds of *Rangifer sp*. in southwestern England are related to the pre-Boreal and come from, among others, Kitley Shelter Cave, Aveline’s Hole, Darent Gravels, Gough’s Cave and King Arthur's Cave [50,51]. In Denmark, reindeer appeared about 12700 cal BC, while the most recent remains of this species are dated to about 8400 cal BC, found at Risbanke site [52]. The above chronology is consistent with the youngest finds of *Rangifer sp*. in southern Sweden (Scania), where the said species spread 1000 years later than in Denmark [53–55]. A different situation can be noted in the northern region of this country, where the oldest reindeer remains come from Kangos Raä site and are dated similarly to the artefact from Gołębiewo [56]. The similar radiocarbon dates were estimated also for the layer containing bones of *Rangifer sp*. at Dumpokjauratj site [57], whereas 700-years younger remains at Kitkiöjavri site [56]. It is possible that one of the oldest reindeer bones from northern Sweden known to date come from two sites dated to about 8600 cal BC near Aareavaara. However, the species remains uncertain [58,59].

In the Baltic States, similarly as in other parts of the European Plains, the youngest finds of *Rangifer sp*. bones are dated to the pre-Boreal [60]. In Latvia, the age of three beams [54] was estimated by radiocarbon dating as the period between the turn of the 12^th^ millennium and the end of the 11^th^ millennium cal BC. A series of Late-Glacial dates was estimated in Latvia for plant remains excavated in a layer containing reindeer bones at a site nearby Tetele. From among 7 finds of *Rangifer sp*. antler in Lake Lubãns, the age of the youngest one was estimated at about 9500 cal BC [54,60]. Possibly, a harpoon from Zvejnieki II site is a much older find, as its age was estimated as mid-9^th^ millennium cal BC. However, the view that reindeer antler was used for making this product remains solely a suggestion [60]. A number of stray finds of *Rangifer sp*. bones is known from the area of Estonia. Finds in Lake Vasula are associated with Late Glacial, whereas the youngest excavated finds of this species are two antler beams from Lake Kunda dated to the turn of the Holocene [54,60]. It is difficult to refer to the actual age of the reindeer bones from Kunda Lammasmägi site, where the oldest obtained radiocarbon date was 9330±120BP (UA-3005). Nevertheless, it was obtained for an elk bone, while reindeer remains might be older [54].

The lack of finds that would indicate migration of this species in the Early Holocene to southern Finland [60] means that the Estonian remains define the northern limit of distribution of the periglacial reindeer, most likely determined by severe post-glacial environmental changes about 9000 years BP [53]. At that time, Finland was an archipelago of islands, with only its eastern part being largely over the water surface. The drying areas of southern and central parts of the country were taken over by vegetation and forest habitats in an exceptionally short time and only the area of northern Lapland constituted a habitat friendly to the reindeer [53]. This species could colonise the area of the present-day Finland in two ways [53,54,61,62]. The earliest finds from the northern part of the country most likely represent the descendants of the Central European population (*Rangifer tarandus tarandus* L.), which spread north along the coast of Norway. It is also probable that a migration from the East occurred simultaneously, along the Kola Peninsula [53,63], which is supported by results of genetic research [64,65]. The other southern trail of colonisation led most likely about 7500 years ago from Siberia and was a consequence of the reindeer population adapting to the forest habitat (*Rangifer tarandus fennicus* Lönnb.) [48,53].

Until recently, the oldest reindeer remains in Finland were those excavated in Enontekiö and Inari in the northern part of the country and were relatively young, as dated to only about mid-7^th^ millennium cal BC. In the central and southern Lapland, the oldest bones of this species were found in Taivalkoski and Posio, while their age was determined as late 6^th^/the first half of the 5^th^ millennium cal BC. In south-eastern Finland, the youngest archaeological context for reindeer were determined by finds related to Early Asbestos Ware dated to 5500–5000 BP [53,54,66]. This situation changed considerably as a result of recent discoveries. A series of dates was determined for Sujala site in northern Lapland with *Rangifer sp*. prevalent in the bone remains. The youngest of the dates indicate the turn of the 8^th^ millennium cal BC [61,67]. In general, the same dates were obtained for a burnt reindeer bone found at Malmio 1 site about 200 km south-east [68]. The date contextually determined for the reindeer bone at Løkvika site in Norway by the Barents Sea indicates the turn of the 9^th^ millennium cal BC [63]. In southern Finland, the age of the above mentioned ornamented item made of reindeer antler found at Lepaa site [31] is estimated at 6300 cal BC.

The *Rangifer sp*. remains from Scandinavia and the North European Plains cannot be assessed in isolation from those from the present-day western Russia. Few reindeer bones dated to the End-Pleistocene period and the Early Holocene were found, i.a., in north-western part of the country, at an Olenii Ostrov cemetery on Lake Onega in East Karelia [32]. The age of human remains, that provide context for the said remains, is estimated at 7300 and 7700 BP [69]. These were also found in layers at Zamostje 2 site near Moscow [34,35]. The chronology of these finds is similar (about 7300–7200 cal BC, calibrated). The age of layer 4 at Vylys Tom 2 site on River Izhma [70,71], containing *Rangifer sp*. remains, was estimated at first half of 8^th^ millennium cal BC. Finds excavated at Parch 2 site on River Vychegda are older and younger than the artefact from Gołębiewo [72,73].

These data suggest that reindeer were present in the area of the present-day Central Europe, southern Scandinavia and the British Isles not later than until the pre-Boreal Holocene, while in the Boreal period they appeared solely in northern Scandinavia (see [48,50,54,74]). In the time when the artefact found in Gołębiewo was produced, reindeer could have been encountered in Lapland (probably also in the neighbouring North Karelia) in the area north of the line that runs from the base of the Kola Peninsula to the contemporary north end of Ancylus Lake (in general, parallel 66°/ the Arctic Circle), on the coastline of that reservoir nearby a melting ice sheet in north-eastern Sweden and Russia, in the north-eastern part of the East European Plains (Fig 6). Although no reindeer remains contemporary with the Gołębiewo artefact have yet found from the area between north Finland and Norway, and the area of Vylys Tom 2 site and Parch 2 site in Russia (including the Kola Peninsula), they are quite likely to be found in the future, as suggested by, among other things, the above mentioned genetic studies.

### Geographic origin of *Rangifer tarandus* antler excavated in Gołębiewo

The area considered as the possible origin of the raw material used for making the discussed *bâton percé* is located at a considerable distance to the place where it was found, which puts to question the possibility of a fast transport of the item, whereas the very radiocarbon dating excludes the possibility that the fossil antler has an earlier chronology and was re-used. In the attempt to explain the geographic origin of the raw material used for producing the artefact, isotopic studies were performed, including δ^18^O analysis. The said method allows one to identify the geographic region with habitats of both single animals and entire groups (i.a. [75–79]). The analysis of stable oxygen isotopes in the osseous tissue is used also for tracing seasonal migration of animals and people (i.a. [80–83]), the migration of individuals between groups and reconstructing the settlement process (i.a. [77,84,85]).

In order to provide an answer where did the antler used for making the analysed *bâton percé* come from, the results of the oxygen isotope analysis performed on a bone sample were compare with the range of the δ^18^O isotopic variability of precipitation water of different natural habitats in areas stretching from Northern Poland to Scandinavia. Importantly, the oxygen isotope ration in precipitation water is highly correlated to the composition of environmental water consumed by the animal [18,86].

**Fig 7:**
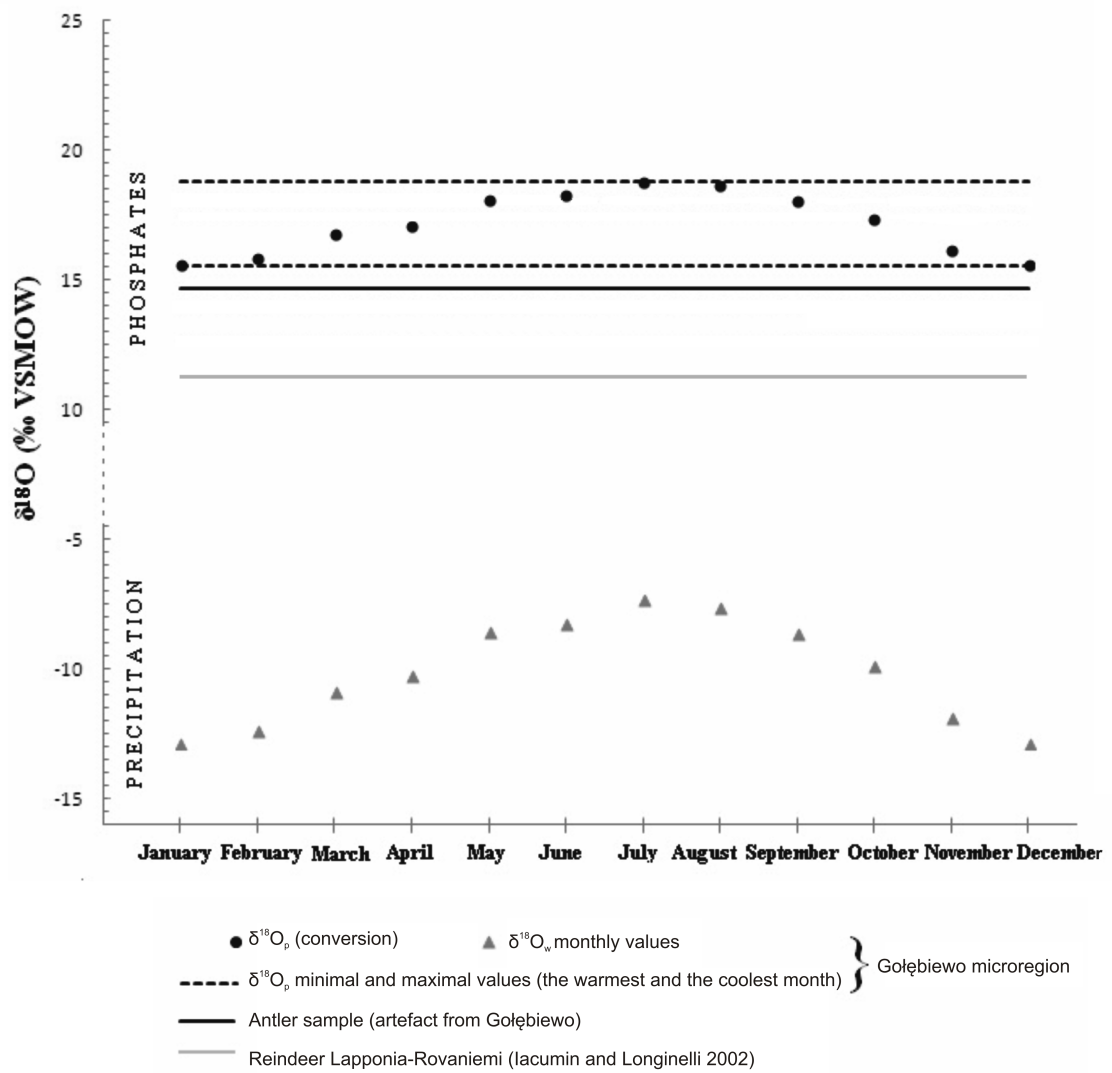
Monthly values of the isotopic composition of precipitation water for Gołębiewo converted to δ18Op concentrations and also δ18Op antler samples from the artefact found in Gołębiewo. The chart presents also the average value of δ18Op for reindeers from the Arctic Circle estimates based on the data derived from the study by Iacumin and Longinelli [16].

The δ^18^O_p_ value for the sample of the analysed artefact (δ^18^O_p_ = 14.69‰) was found to be below the local range determined based on the value of the isotopic composition of precipitation water in Gołębiewo (Table 3, Fig 7). The difference is about 1 per mil in regard to the minimum value, determined for precipitation water from winter months, suggests that the analysed sample does not come from the location where it was found.

**Table 3:** Oxygen isotope values for the analysed sample and environmental water (δ^18^O_w_) from over ten locations representing the regions of Northern and North Eastern Europe, converted to isotopic values of bone phosphates with conversion (δ^18^O_p (eq. conversion)_) equation no. 5 [18].

Site	Country	δ18Ow [‰, VSMOW]	δ18Op (eq. conversion) [‰, VSMOW]		
			$\bar{x}$	min	max
Gołębiewo, artefact	Poland		-14.69		
Gołębiewo	Poland	-10.05	15.46	15.26	15.62
Espoo	Finland	-11.73	16.21	16.13	16.27
Kuopio	Finland	-13.84	14.96	14.70	15.17
Talvivaara	Finland	-12.40	14.36	14.01	14.64
Kuusamo	Finland	-12.80	14.13	13.75	14.43
Gothenburg	Sweden	-8.46	18.13	17.97	18.34
Stockholm	Sweden	-10.16	17.13	17.08	17.19
Umeå	Sweden	-12.40	15.81	15.68	15.92
Rickleå	Sweden	-12.44	15.79	15.65	15.89
Taastrup	Denmark	-10.10	17.16	17.11	17.23
Lista	Norway	-6.65	19.19	18.91	19.56
Riga	Latvia	-9.59	17.46	17.38	17.57
Tartu	Estonia	-10.78	16.76	16.76	16.77
Vilsandi	Estonia	-9.74	17.38	17.30	17.47
Saint Petersburg	Russia	-11.46	16.36	16.31	16.41
Arkhangelsk	Russia	-13.38	15.24	15.01	15.41
Kandalaksha	Russia	-14.10	14.81	14.53	15.03

**Fig 8:**
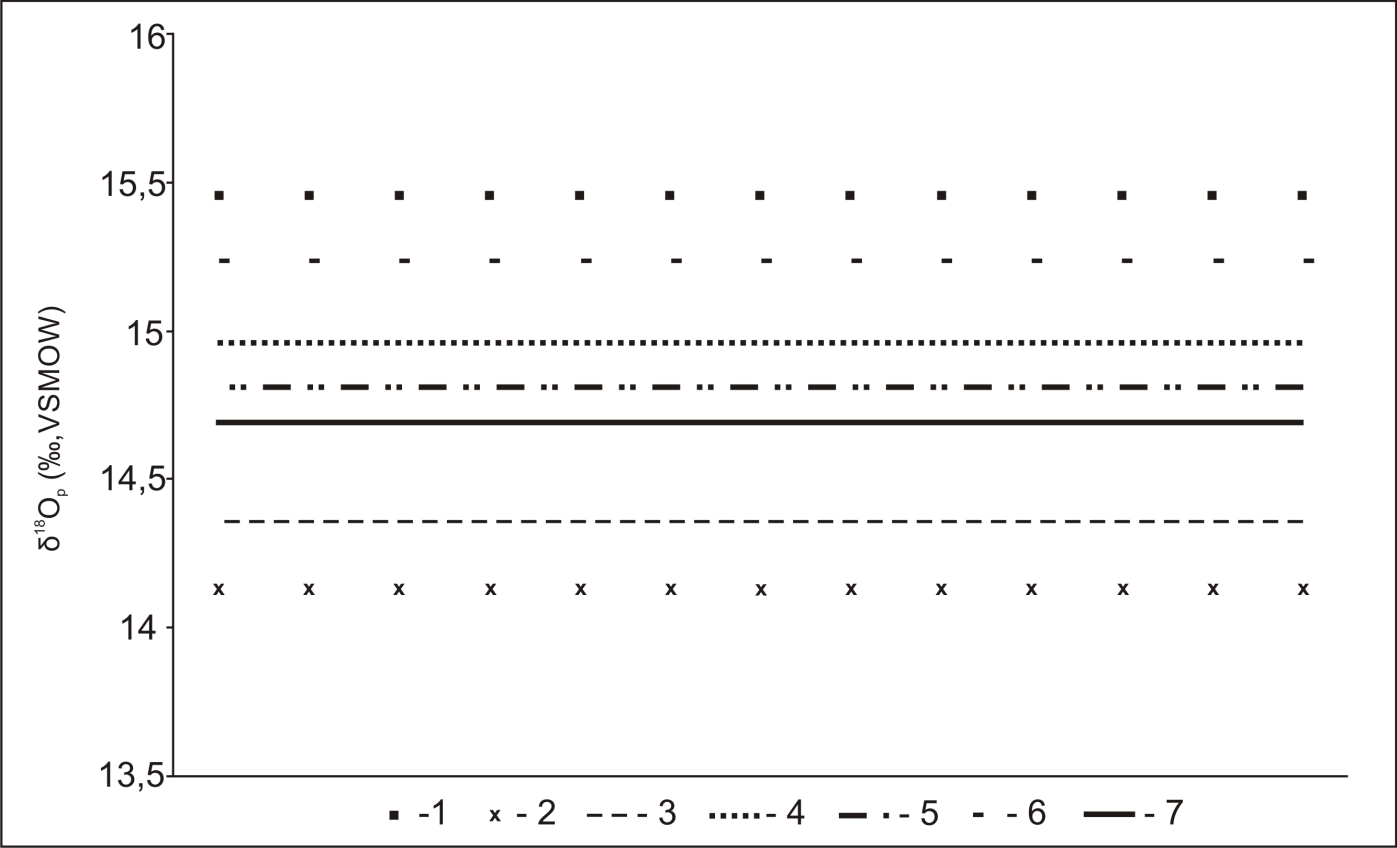
The value of isotopic composition of the antler sample from the artefact found in Gołębiewo in relation to oxygen delta values in precipitation water in Gołębiewo and selected macroregions that best correspond to the value obtained for the sample. Regions: 1 –Gołębiewo, 2 –Kuusamo, 3 –Talvivaara, 4 –Kuopio, 5 –Kandalaksha, 6 –Arkhangelsk, 7 –the artefact from Gołebiewo.

The verification of the hypothesis about the origin of the animal the antler of whose was used for making the analysed artefact from Scandinavia or north-western Russia, required collecting and comparing data on the isotopic composition of environmental water in over ten macroregions of Finland, Sweden, Norway, Denmark, Estonia, Latvia and Russia. Table 3 presents a comparison of the isotopic data (the IAEA-GNIP database) [15] for precipitation water from 17 locations representing different macroregions of North and North Eastern Europe, converted to δ^18^O_p_, using the conversion equation 5 from the study by Daux et al. [18]. The computed oxygen isotope values were compared to the data on δ^18^O_p_ obtained for the artefact from Gołębiewo. The isotopic composition of bone phosphates of the examined sample (14.69‰) falls outside the value determined for: Sweden (15.79–18.13‰), Norway (18.91–19.56‰), Denmark (17.11–17.23‰), Latvia (17.38–17.57‰) and Estonia (16.76–17.38‰), furthermore, as presented in Fig 8, it is similar to the value obtained for areas located north and east of the Kuopio macroregion.

It is known that three of the environmental elements may affect the isotopic composition of drinking water, including surface water, groundwater and soil moisture. The composition of surface water and groundwater is most affected by precipitation, with the highest variability of the isotopic composition pertaining to flowing waters (rivers), in which δ^18^O is modified with each consecutive tributary in a new territory [87]. Consequently, the verification of the hypothesis on the possible origin of the discussed antler from the area of Lapland required the δ^18^O_p_ value for the sample from (14.69‰) to be compared with the value of the oxygen isotope composition for surface water, which reflects the local isotopic composition of the areas in southern Finland (mean δ^18^O_p_ = 15.64‰) and northern and eastern parts of this country (δ^18^O_p_ = 14.53‰)–[17]. The environmental content of oxygen isotopes was estimated using the conversion equation 5 from the study by Daux et al. [18]. As presented in Fig 8, the composition of stable oxygen isotopes in the artefact from Gołębiewo is close the δ^18^O_p_ value of those obtained for the northern and eastern Finland, which seems corresponding with the data provided above.

The interpretation of the geographic origin of the sample requires the local oxygen isotope composition to be estimated in an environment based on the δ^18^O_p_ value obtained for the simultaneous animal material derived from the very same archaeological site. Though no other animal bone remains were found in Gołębiewo, the data from the study by Iacumin and Longinelli [16] were used, in which the authors determined the isotopic composition for bone phosphates, i.a., for several *Rangifer tarandus* individual animals. Considering the correlation between the δ^18^O_p_ value of the environment and the outcome of the animal bone analysis, the δ^18^O_p_ values of the sample taken from the artefact from Gołębiewo were referred to the study results presented in the mentioned paper.

The mean δ^18^O_p_ value for the reindeer living in Lapland-Rovaniemi is 11.25‰ [16]. Due to a difference in the δ^18^O_p_ values in the analysed sample and the bones of these animals (Fig 7) it should be concluded that the antler used for making the examined artefact does not come from the area located west of the line presented in Fig 6, which designates the range of oxygen isotope values corresponding with the value obtained for the analysed sample.

The result of the isotopic composition of the precipitation water from the area of Gołębiewo suggests a non-local origin of the *bâton percé* found there. The comparison of data presenting oxygen isotope values in the considered macroregions of Finland, Sweden, Denmark, Norway, Estonia, Latvia and Russia, as well as the δ^18^O_p_ value determined for reindeer from the area of the Arctic Circle suggest that the studied antler comes from an area north and east to the Kuopio macroregion up to the Kola Peninsula on the Kuopio-Talvivaara-Kuusamo-Kandalaksha-Arkhangelsk line (Fig 6). Considering the finds of *Rangifer tarandus* remains known from this region of a chronology close to the age of the artefact from Gołębiewo, it should be concluded that its northern part is most probable (the southern end of Lapland/North Karelia and the area at the base of the Kola Peninsula), since no remains of representatives of this species with a relevant chronology have been found so far in its western and southern parts, which in accordance with present scientific knowledge is highly unlikely. In the regard of the situation in south-eastern part of the designated region (the coast of the White Sea), where, as noted above, their presence is probable.

Drawing conclusions based on the data concerning the oxygen isotope values from phosphate finds support in the content of stable carbon and nitrogen isotopes of collagen. The carbon and nitrogen content (%C, %N) of the specimen, and its atomic carbon-to-nitrogen (C:N) ratio are within the range of acceptable collagen quality values [88]: %C = 42.3%; %N = 14.7%, and C:N = 3.4. The specimen from Gołębiewo exhibits a δ^13^C value of -20.6‰ and a δ^15^N value of 3.5‰.

**Fig 9:**
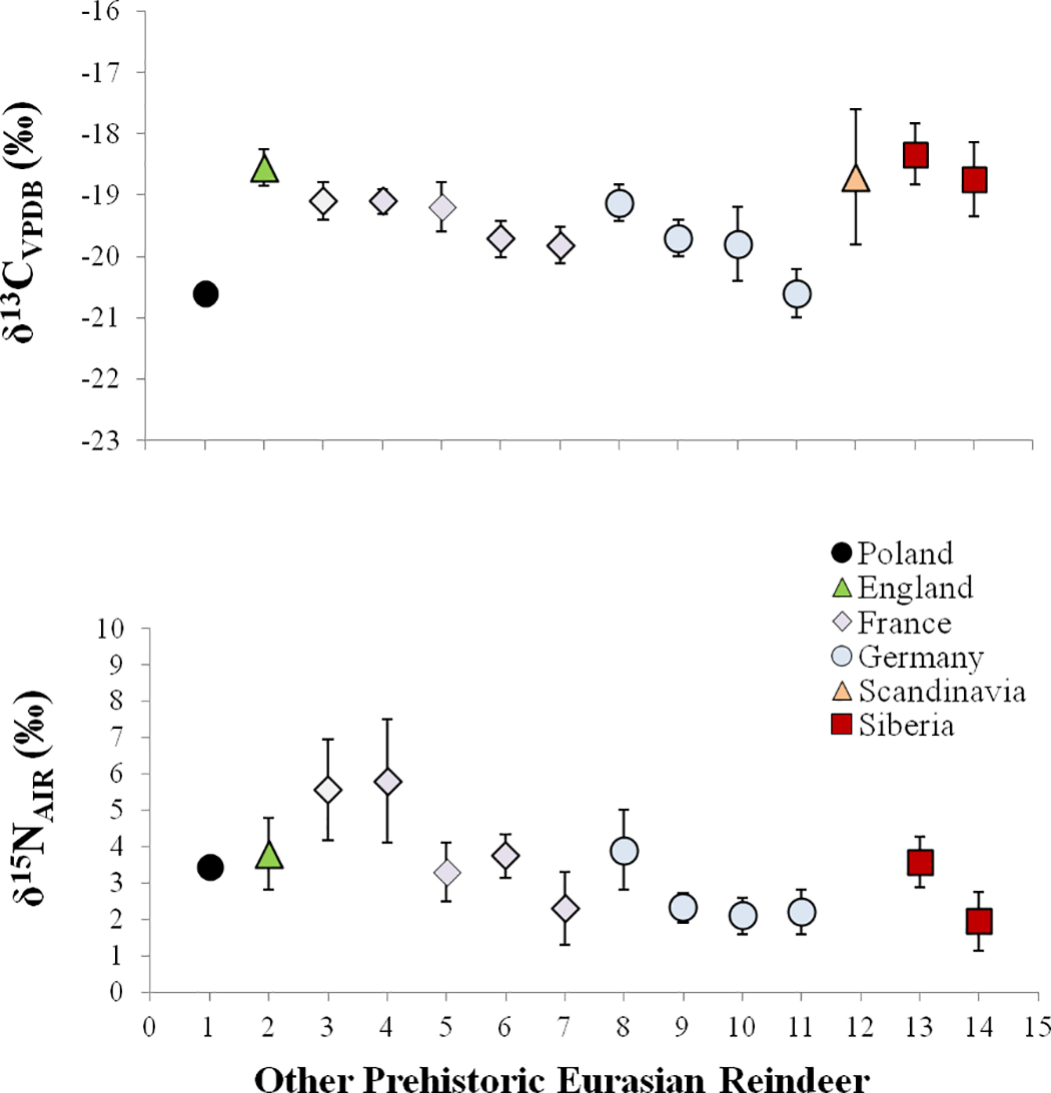
Stable carbon and nitrogen isotopic comparison between the reindeer specimen in the present study, from North-Central Poland, with other reindeer (all prehistoric Eurasia) reported as means±one standard deviation (1 –present study; 2 –after [89]; 3 and 8 –after [90]; 4 –after [91]; 5 –after [92]; 6 –after [93]; 7 –after [94]; 9 –after [49]; 10 and 11 –after [95]; 12 –after [52]; 13 and 14 –after [96]).

In comparison to other *Rangifer* from elsewhere in Eurasia, the δ^13^C value of this particular specimen is relatively low, suggesting the habitat of the reindeer in the present study was more forested than those of reference samples, which are located in England, Germany, Siberia, France, and Scandinavia (Fig 9) [49,52,89–96]. To our knowledge, no other archaeological reindeer collagen stable isotope values are available from habitats in Poland (cf. [97]). However, deer (*Cervus elaphus; Capreolus capreolus*) reported by Reitsema et al. [98] and Pokutta [99] from Poland (medieval and Bronze Age, respectively) exhibit δ^13^C values of approximately -22% to -23‰, lower than the reindeer in the present study. However, this could be expected even when *Tarandifer*, *Capreolus*, and *Cervus* co-exist in the same ecogeographic region, as reindeer will tend to eat lichens when available, unlike deer, allowing them to have slightly higher δ^13^C values despite shared territory.

While δ^13^C and δ^15^N data from a single specimen cannot on their own resolve the question of ecogeographic origins, it is clear that the δ^13^C value of this specimen is not as high as for tundra-dwelling reindeer. Instead, the relatively low δ^13^C value for the reindeer in the present study suggest a forested habitat with plentiful vascular plants. The δ^15^N ratio in the present study is higher on average than the comparative specimens, suggesting a less cold climate and more open nitrogen cycling in soils. Together, these data suggest the reindeer in the present study lived in a forested habitat, probably not in the arctic or subarctic. Similarly as in the case of the oxygen isotope analysis, the result of the analysis of stable carbon and nitrogen isotopes excludes the northernmost part of Europe beyond the Arctic Circle from consideration. However, it does not eliminate the area of North Karelia, which used to be covered with an open forest as early as from ab. 9000 cal BC according to the results of palynological analysis [100,101]. Unfortunately, the information pertaining to the isotopic composition of reindeer bones from this region is unavailable. The only data on the δ^13^C isotope content come from the area of northern Finland and their value is by 3 to 7 pre mille higher than that obtained for the artefact from Gołębiewo [68]. It should be noted that the analysis was not performed on burnt bones, whose carbon and nitrogen isotope content is higher and reaches 4 to 6 ‰ [102,103]. Thus, it can be assumed that the biogenic value of such bones ranged from 19 to 22 ‰, and was similar to the result for the analysed item.

### Was contact between the Mesolithic communities of Central Poland and Scandinavia possible? Discussion

The findings of multidisciplinary studies are encouraging to reflect on the meaning of the *bâton percé* from Gołębiewo for the Mesolithic hunters who used it, and the reason why the raw antler material was transported from such a great distance in what seems a short time. We are most certainly not dealing with a system of organised exchange in the type of Kula in Melanesia [104], which is suggested by a single character of the find. Similarly to the reindeer bone from Rottenburg-Siebenlinden 1 site [48] the exchange of this object is probably a result of a one-time excursion not necessarily as far north as northern Scandinavia, and the travel and exchange could be motivated by other reasons for inter-group contact besides trade itself, e.g., *rites de passage*, or a side-effect of a social contact between groups [1,105]. Naturally, it cannot be ruled out that on a certain part of the journey between the location of the origin of the antler and the region where the *bâton percé* was made and used it constituted an object of a regular exchange of a shorter territorial coverage. There are many factors pointing to flow of ideas or artefacts along pathways and networks leading to present-day Poland (Fig 6).

The entire area of Scandinavia and the Baltic States lies in the area where the conical core pressure blade concept, originating in the Butovo Culture in the Volga basin, was spread [56]. In this respect, direct contact can be evidenced by, for example, the presence of Pulli-type points in Poland and in the Upper Volga basin typical of the Kunda Culture from the Baltic Republics [56]. In this part of the Early-Holocene Europe, similar trails were used in the spreading of the idea of the manufacturing of slotted bone tools and the shaft-wedge-splinter technique used in bone processing [106,107]. Here, a number of elements point to the existence of relationships that fostered the exchange of ideas. Though the places of origin of flint materials in Finland are in the southern and south-eastern part of the country, the flint used in this area could have been imported also from Estonia, Russia, Belarus, Lithuania or even Poland [68,108]. The presence of features characteristic of post-Swiderian technologies on sites like Sujala in northern Finland or Fállegoahtesajeguobba in Norway, considering the lack of proper stone materials between them and areas where the technologies originated, indicates that excursions organised on this route (ab. 1200 km) could not have taken longer than one generation. Otherwise, providing the offspring with vital knowledge would have been impossible [56,61]. On the other hand, in western Scandinavia, interregional contacts are manifested by the flow of blade insets (elements of slotted points, originally) from the south and the west of the said area to central Sweden [105].

**Fig 10:**
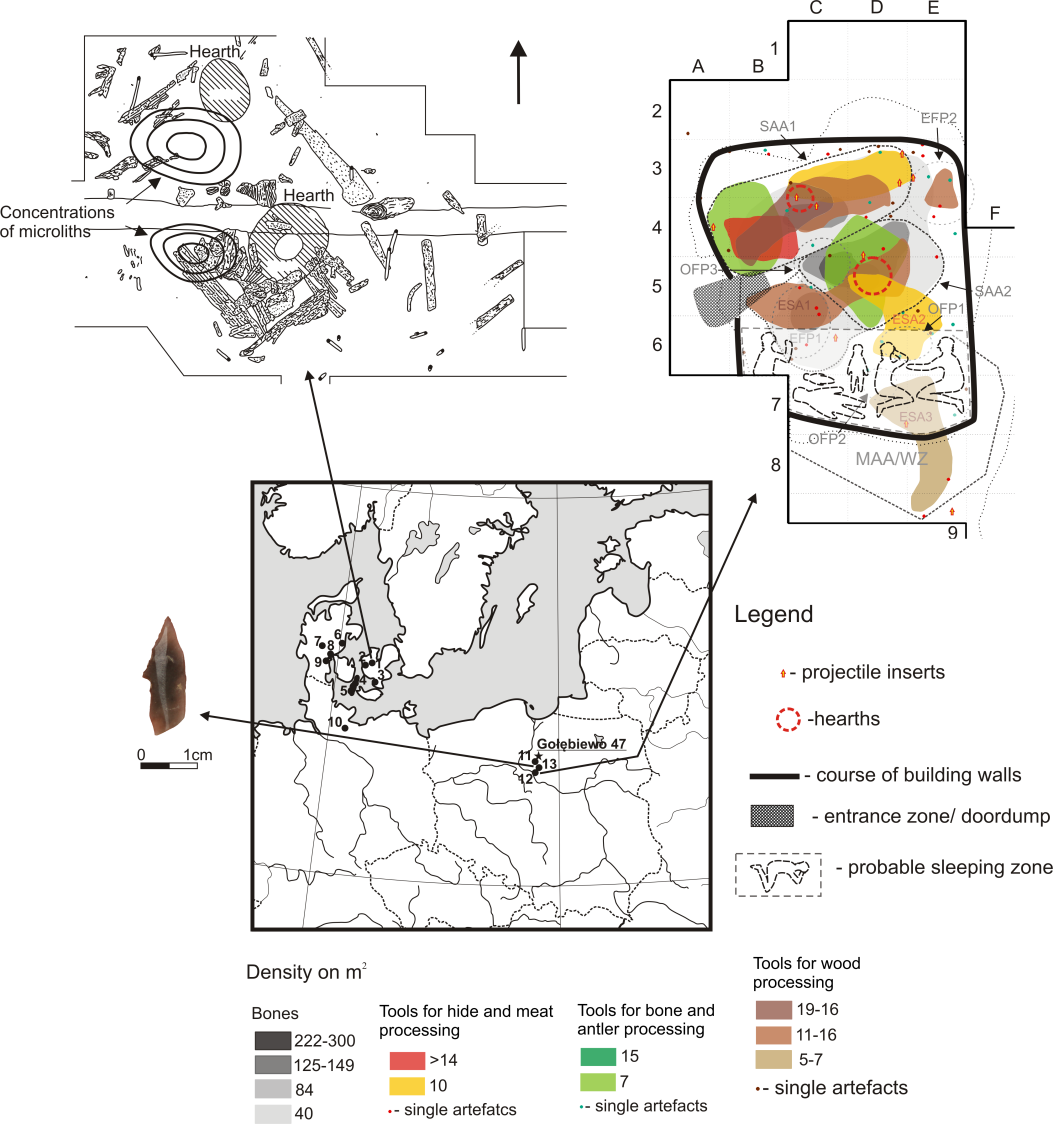
Location of sites with Ulkestrup I residential structures with the structures at Ulkestrup I (after [109]) and Sąsieczno 4 (after [8]) sites depicted. Sites: 1 –Ulkestrup I, 2 –Mullerup, 3 –Sværdborg II, 4 –Flaadet, 5 –Magleby Nor, 6 –Rude Mark, 7 –Bøllund, 8 –Stallerupholm, 9 –Svanemosen 28, 10 –Duvensee, 11 –Ludowice 6, 12 –Sąsieczno 4, 13 –Paliwodzizna 29.

Although as yet there has been no evidence for the existence of direct contact between the Mesolithic communities of the Chełmno-Dobrzyń Lake District in Central Poland, and the Scandinavian region where the antler described here most likely comes from, it is possible to distinguish several preconditions for a high likelihood of indirect contacts. There are considerable similarities between flint processing techniques and types of insets used between the area of the present-day Poland, Bornholm and the adjacent areas of Denmark and Sweden [56]. Support for a relationship between these two regions are also the structure, spatial orientation and internal organisation of the shallow pithouse discovered recently at Sąsieczno 4 site and (to a central extent) shelter from a western habitation at Ludowice 6 site (both located in the Lake District of Chełmno and Dobrzyń –Fig 10)–[7,8]. These are closely analogous to the two-family residential structures of type Ulkestrup I known from the Maglemose sites in the area of Denmark and southern Sweden (i.a. Ulkestrup I, Sveardborg II, Flådet or Duvensee W.6 –[109,110]. At Ludowice 6 and Paliwodzizna 29, nearby barbed points/*hullingspetsen* were found, which are significant in the view of the discussed issue (Fig 10). The said finds are implements typical of Bohuslän province in south-western Sweden [111] and are also known from other sites situated in this part of Scandinavia, e.g., Ringsjöholm in Scania [112]. Products of this sort were also excavated at other Mesolithic sites in north-western Poland [113]. The unique stone quarries identified at Ludowice 6 site based on non-flint materials, mainly quartz sandstone and red porphyry [114,115] may prove the flow of ideas between the Lake District of Chełmno and Dobrzyń and eastern Scandinavia. Similar collections are known mostly from the area of Sweden, Norway, Finland and Estonia (i.a. [63,116–120]).

All the mentioned finds and the results of multidisciplinary studies conducted on the *bâton percé* from Gołębiewo constitute a strong precondition for the existence of a relationship between Early-Holocene hunter-gatherer communities in the Lake District of Chełmno and Dobrzyń, as well as the groups inhabiting Scandinavia including its north-eastern part. However, determining the nature of these contacts and their scale calls for further multifaceted research. Here, it is worthwhile to estimate the time necessary for the antler to reach Gołębiewo from North Karelia. In this respect, the situation recalled Sørensen et al. [56] derived from a study by Mowat [121] can serve as a reference. That particular paper provides a description of an expedition taken by Samuel Hearne from Prince of Wales on the Hudson Bay to Coppermine River on the coast of the Arctic Sea (ab. 1,700 km) in the 18^th^ century. Hearne walked the distance accompanied by Chipewyan Indians across uneven lands, fishing and hunting on the way, using simple technology. He reached his destination in 223 days, which gives an average of 7.2 km per day. Considering this average and the course of the routes the bone material could have been transported on from North Karelia to the area nearby Gołębiewo (Fig 6), the estimated duration of the shortest one (across Finland) is 277 days or 486 days in the case of travelling along the Norwegian coast. Therefore, it can be stated that the transport of a relatively small antler beam at such a distance was possible in the Early Holocene even in a relatively short time (up to, or exceeding, ten years). Obviously, this would require cultural conditions favouring interregional mobility, which is highly likely as claimed by the evidence for the existence of a relatively free flow of ideas and items in the region.

## Conclusions

The *bâton percé* from Gołębiewo is a significant item representing the art of the Early-Holocene hunter-gatherers with a probable zoomorphic stylisation. More importantly, though, being currently the youngest reindeer bone artefact in the European Plains and southern Scandinavia, it likely constitutes an evidence for interregional contact in the European Mesolithic, characterised by a nature and range that go beyond the schemes validated by current knowledge. The route taken for transporting the *Rangifer tarandus* antler from nearby North Karelia to Central Poland, and the motive for transporting it, remain impossible to determine conclusively. However, the obtained results are the first direct evidence for the flow of goods between hunter-gatherer groups in the Early Holocene at such a great distance. Together with findings provided by other authors, the presented data serve as a contribution to the further multifaceted search for traces of idea and item exchange in the Mesolithic within the area of North Eastern Europe.
